# Mechanical Energy Absorption Ability of Titanium-Based Porous Structures Produced by Various Powder Metallurgy Approaches

**DOI:** 10.3390/ma16093530

**Published:** 2023-05-04

**Authors:** Pavlo E. Markovsky, Jacek Janiszewski, Oleksandr O. Stasiuk, Dmytro G. Savvakin, Denys V. Oryshych, Piotr Dziewit

**Affiliations:** 1 G.V. Kurdyumov Institute for Metal Physics of N.A.S. of Ukraine, 36, Academician Vernadsky Boulevard, 03142 Kyiv, Ukraine; stasiuk@imp.kiev.ua (O.O.S.); savva@imp.kiev.ua (D.G.S.); deni@imp.kiev.ua (D.V.O.); 2 Faculty of Mechatronics, Armament and Aerospace, Jarosław Dąbrowski Military University of Technology, 2, Gen. Sylwester Kaliski Str., 00-908 Warsaw, Poland; jacek.janiszewski@wat.edu.pl (J.J.); piotr.dziewit@wat.edu.pl (P.D.)

**Keywords:** porous titanium, mechanical properties, high strain rate testing, direct impact Hopkinson pressure bar technique, microstructure influence, deformation mechanism, energy absorption

## Abstract

Porous materials are very efficient in absorbing mechanical energy, for instance, in combined armor, in order to improve the anti-ballistic protection characteristics. In the present study, porous titanium-based structures were manufactured via three different powder metallurgy methods using titanium hydride (TiH_2_) powder, which provided activated sintering, owing to dehydrogenation. The emission of hydrogen and shrinkage of powder particles on dehydrogenation also added an additional potential to control the sintering process and create desirable porosities. TiH_2_ powder was sintered with additions of NaCl or ammonium carbide as pore holding removable agents, while highly porous Ti-Al structures were formed via liquid phase reactive sintering of TiH_2_ and Al powders. The microstructures and porosities of sintered dehydrogenated titanium and Ti-Al structures were comparatively studied. Mechanical characteristics were evaluated using compression testing with strain rates varying from quasi-static to high levels. The resonant frequency method was also employed to determine damping parameters and elastic modulus of these materials. All testing methods were aimed at characterizing the energy-absorbing ability of the obtained porous structures. The desired strength, plasticity and energy-absorbing characteristics of porous titanium-based structures were assessed, and the possibilities of their application were also discussed. Based on the obtained results, it was found that porous titanium materials produced with the use of ammonium carbonate showed promising energy absorption properties.

## 1. Introduction

Metallic materials with controlled porosities are characterized by a number of unique physical and mechanical properties, due to which they are widely used in various fields—from filters to impact energy-absorbing systems [1,2,3,4]. A wide range of different technological approaches are used to obtain such materials: foaming of liquid metals, laser printing with metallic powder, powder metallurgy method including employment of removable space holders [2,3,4,5,6]. The last method, powder metallurgy with space holders, is the simplest and most versatile, allowing one to obtain materials with porosities of more than 80%, high specific strength, and sufficient strain values when tested with different deformation rates. Concerning titanium and titanium-based porous materials, early works were devoted to the production of porous titanium using NaCl as a space holder [7,8]. These studies were carried out either on hydrogenated/dehydrogenated titanium powder [7] or globular powder [8] sintered at relatively low temperatures (780 °C), which correlated well with the properties (strengths) of cancellous bones and was promising for use in implants. The works in which not only NaCl was used as a space holder together with aluminum powder ensuring the formation of highly porous intermetallic compounds of the Ti-Al system should also be noted [9,10,11]. Latter works can be mentioned separately because they describe a technological approach uniting an exothermic reaction between Ti and Al powders as a method of material synthesis with the application of calibrated NaCl crystals as a removable space holder to form the specific porosity. The mechanical behavior of porous materials is determined with specified parameters of porosity, including total volume content, size of pores, and uniformity of their distribution. Thus, to achieve desirable strengths, ductility levels, and dumping capacities, the parameters of pore structures should be strictly controlled. The aim of the present work was the fabrication of highly porous titanium-based materials in three different methods, employing earlier developed powder metallurgy approaches based on the employment of titanium hydride instead of pure titanium [12] to obtain desirable porous structures and investigations of the mechanical behaviors of produced materials under conditions of quasi-static and dynamic compression in order to evaluate their potential for energy absorption.

## 2. Materials and Methods

**Figure 1:**
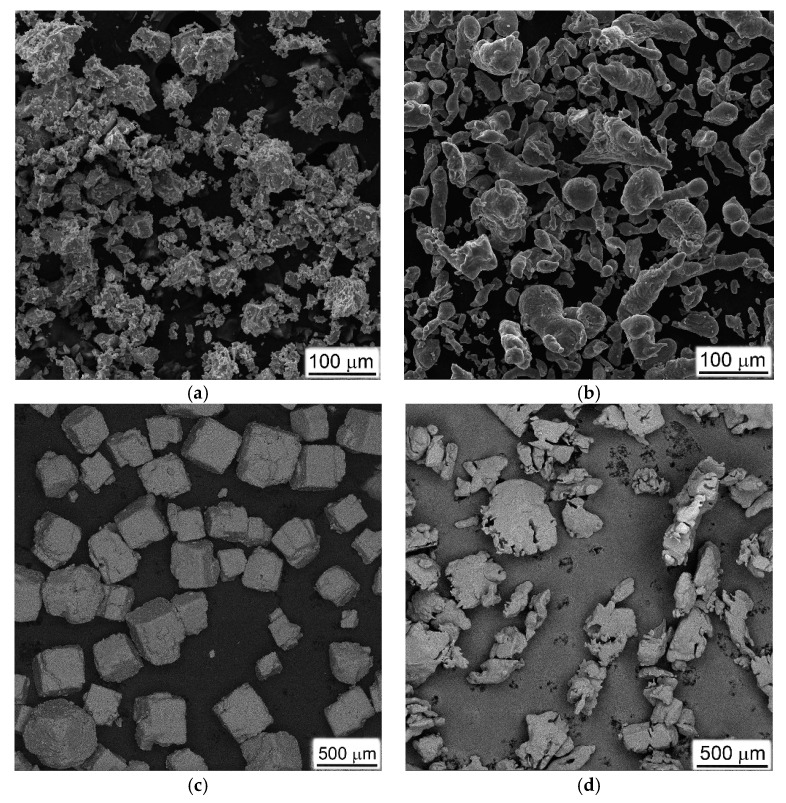
Microstructures of initial powders: (**a**) TiH_2_, (**b**) Al, (**c**) calibrated NaCl, and (**d**) ammonia carbonate (space holders), used for preparation of porous Ti. SEM, (**a**–**c**) SE, (secondary electron image) (**d**) BSE (back-scattered electron image).

Three different powder metallurgy approaches employing titanium hydride TiH_2_ as raw powder [12,13] were tested in the present study to produce porous titanium-based materials. These different titanium-based porous material fabrication methods were used for comparison with each other in terms of ease of manufacturability and energy absorption efficiency. To achieve increased porosities, titanium hydride powder with a particles size of <100 µm (Figure 1a) was blended with either aluminum powder (Figure 1b), sodium chloride (Figure 1c), or ammonia carbonate (NH_4_)_2_CO_3_ (Figure 1d). Three types of corresponding blends (Table 1) were compacted at room temperature at 150 MPa pressure and sintered using specified regimes to achieve dehydrogenation of titanium hydride, consolidation of titanium particles, and removal of space holder agents. Porous commercially pure titanium (CP-Ti) was produced with two space holder methods. The first of them used a sintered blend of TiH_2_ powder and calibrated NaCl crystals as removable space holders after sintering by water dissolution [13]. Sintering was carried out in two stages: the preliminary at 1000 °C under vacuum for the dehydrogenation of TiH_2_ and initial joining of titanium particles, after which the salt was washed out using boiled water, and, after drying of samples, they were subjected to final vacuum sintering at 1200 °C (see p. 1 in Table 1). As a result of preliminary experiments, it was established that the amount of NaCl crystals in the starting TiH_2_ + NaCl mixture must have been at least 60% to create open porosity in the sintered material, which was necessary to completely remove the salt in order to obtain a sufficiently consolidated porous material by this method. The second method produced porous CP-Ti using ammonia carbonate (NH_4_)_2_CO_3_) as a removable space holder [14], which could be removed by annealing of green compacts at 150 °C in open air before vacuum sintering (##4–6 in Table 1). Additionally, highly porous Ti-Al materials containing 20 and 30%Al (wt.) were produced using TiH_2_ + Al powder blends. The amount of aluminum was chosen on the basis of preliminary studies in order to obtain the highest porosity—with a higher and lower Al content, this synthesis method caused a porosity decrease. It is well known [15] that the reaction between solid titanium particles and molten aluminum on heating of such blends immediately resulted in the appearance of titanium aluminide phases and intensive pore formation. Despite using TiH_2_ powder instead of titanium powder, this allowed us to realize this reaction in a solid phase and, in such a manner, to avoid pore formation [12,13]. In the present study, heating regimes were specially selected to provide reactions via liquid phase of aluminum and, hence, to achieve enhanced porosities of produced Ti-Al materials. Some details of sintering regimes and resulting characteristics of samples are shown in Table 1. 

**Figure 2:**
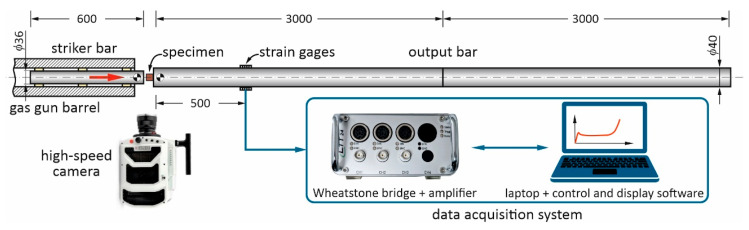
The schematics of the forward DIHPB system used in the present work.

All porous materials were prepared in the form of flat samples with dimensions 12 × 90 × 90 mm. For the elastic properties and damping capacity measurements, specimens with a size of 4 × 12 × 60 mm were cut. Cylindrical samples with gauge lengths and diameters of 10 mm for direct impact Hopkinson pressure bar (DIHPB) and quasi-static compression (QSC) tests were cut by the electric discharge machining technique. The first tests were performed with the installation shown in Figure 2; the second—using the Instron 8802 strength machine. Young’s, Shear moduli, Poisson’s ratios, and Damping capacities of materials were measured with the Resonance-Frequency-Damping Analysis (RFDA) system (IMCE, Genk, Belgium), using the Impulse Excitation Technique (IET), following the ASTM E1876-15 protocol. Material microstructures before and after tests as well as the specimens’ fractures were studied using scanning electron microscope (SEM), Vega 3 (Tescan, Czech Republic), equipped with energy dispersive X-ray (EDX, Bruker, USA) spectroscopy allowing the measurement of the chemical composition of materials. Phase compositions of the specimens and their crystalline structure measurements, including the texture analyses, were performed using an X-ray diffraction (XRD) Ultima IV (Rigaku, Tokyo, Japan) system. The gas content within the sintered specimens was measured using a gas analyzer ELTRA OH900 (Haan, Germany). 

Dynamic compression tests were performed using the DIHPB technique, represented by the forward configuration, which ensured the deformation of the specimens to large strains (up to densification in the case of cellular solids) [16,17]. The schematic drawing of the forward DIHPB set-up is depicted in Figure 2. The used solution of the DIHPB arrangement was similar to the one presented in [18,19]. The currently used laboratory stand consisted of a striker bar with a diameter of 36 mm and a mass of 4760 g, launched from a gas gun and a 6 m long output bar, i.e., two 3 m long pressure bars with a diameter of 40 mm and a data acquisition system. The specimen was put at the front of the output bar, and the 600 mm long striker bar could directly impact the specimen. The striker bar and output bars were made of the same material, i.e., C45 steel with a yield strength and elastic wave velocity equal to 735 MPa and 5140 m/s, respectively. To determine force/stress on the loading surface of a specimen, a pair of strain gages placed 500 mm from the front end of output bar was used to record transmitted signals. The position of the strain gages and the length of the output bar were chosen in order to avoid wave superposition. 

A Phantom V1612 high-speed camera (Vision Research, Inc., Wayne, NJ, USA) was used to measure the shortening of the specimens, the compression rates, and to identify failure modes, as well as to confirm that the striker bar kinetic energy was sufficient to provide a near-constant compression velocity of specimen up to a nominal strain of at least 0.5. High-speed video images were recorded with a resolution of 512 × 208 pixels and a frame rate of 110,000 fps. To ensure high measurement accuracy based on video images, crush test markers and specialized TEMA Classic software version 4.6-002.64 (Image System AB, Linköping, Sweden) were used. The deformation length history of the specimens (shortening) was determined by the subtraction of the displacements between the projectile and output bar. Thus, the corresponding nominal strain could be calculated as performed in quasi-static tests.

## 3. Results 

### 3.1. Characteristics of the Original Structures

**Figure 3:**
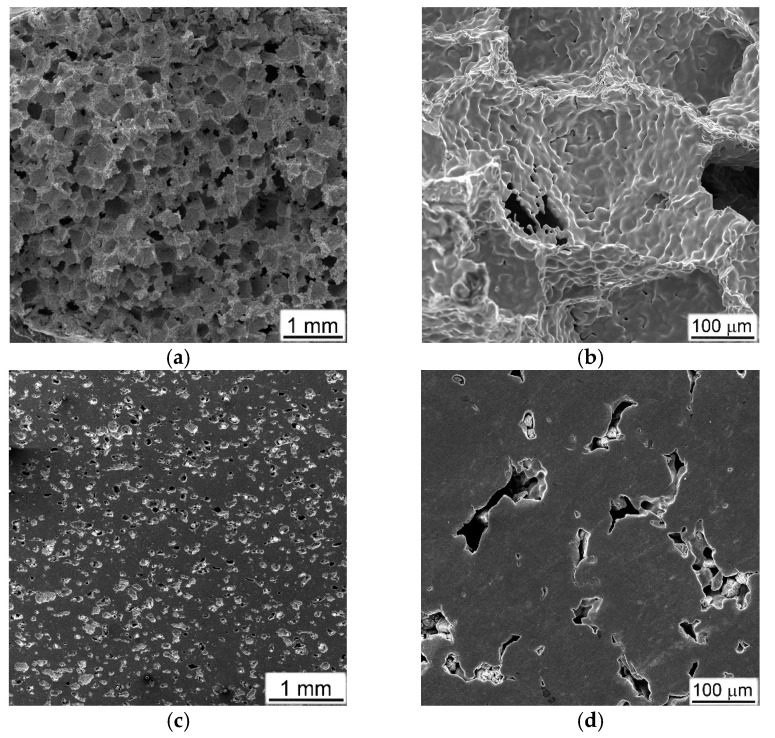

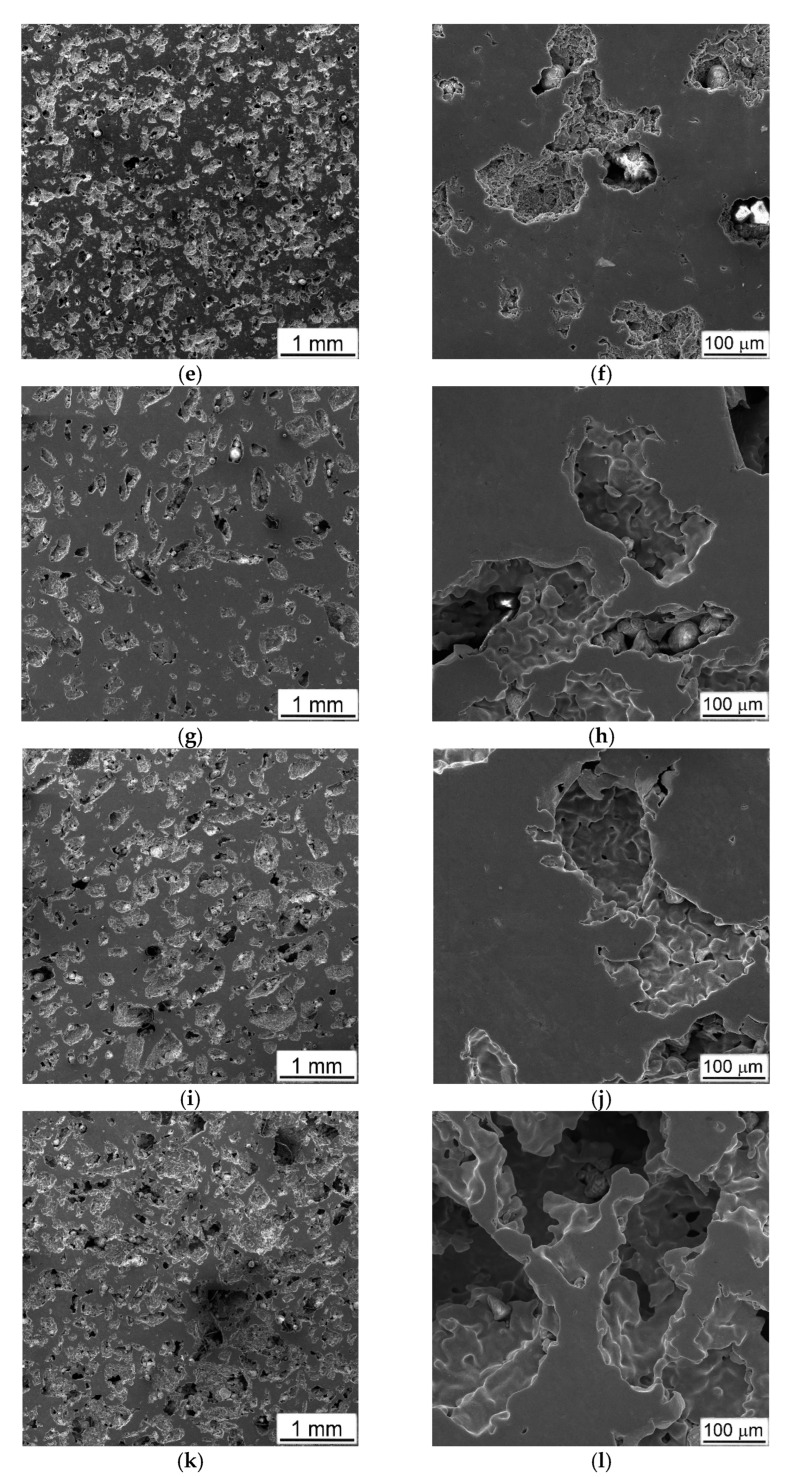
Microstructures of porous materials obtained with different approaches; using space holders: (**a**,**b**) calibrated NaCl (**c**,**d**) 20%Al, (**e**,**f**) 30%Al, and ammonia carbonate (AC) in quantities: (**g**,**h**) 25%, (**i**,**j**) 50% AC, (**k**,**l**) 75% AC. SEM, SE (secondary electron image).

Typical initial microstructures of the studied porous materials are presented in Figure 3. It is clearly seen that all the sintered materials are characterized mostly by open porosities, which were most developed in the case of the NaCl crystals’ employment as space-holding substrates. In this case, the sizes of the formed pores (~200–300 µm) completely inherited the sizes of the initial sodium chloride crystals (Figure 3a,b, and compare with Figure 1c). In addition to large pores in the locations of former NaCl crystals, there is a multiplicity of small (less than 20–30 µm) pores between the consolidated dehydrogenated titanium particles (Figure 3b). This porous titanium is characterized by the highest (71%) porosity (p. 1 in Table 1) that is connected with the peculiarities of using and removing this space holder, noted above in Section 2, related to the necessity to ensure open porosity for the complete removal of salt. The application of aluminum powder to form porous Ti-Al materials (Figure 3c–f, pp. 2, 3 in Table 1) resulted in the smallest (less than 100 µm) average size of irregular shape pores, while the total porosity was increased within 35…42% with an increase in Al content (Table 1). Some chemical inhomogeneity of the local composition with residual not-reacted Ti particles and Ti-Al areas of wide compositions were detected in the microstructure. The application of ammonia carbonate (AC) as a space holder caused wide variations in pore sizes and total porosities (Figure 3g–l, pp. 4–6 in Table 1), depending on the amount of pore holder in the initial blends. 

**Figure 4:**
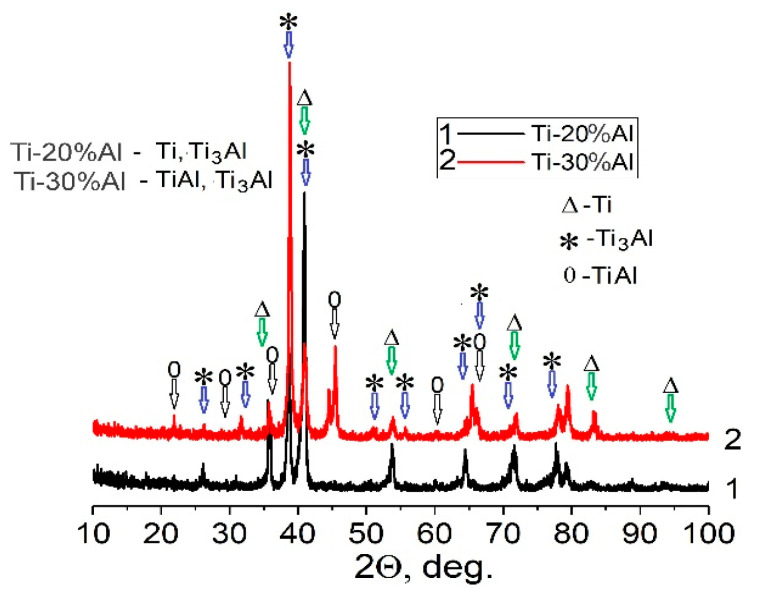
XRD patterns for sintered Ti-20Al (1), and Ti-30Al (2) materials.

It should be also mentioned that porous materials obtained with the employment of both removable agents (NaCl and AC) had single-phase pure Ti compositions, with some minor contamination of the inner surfaces of the pores, with salt residues in the first case and oxides and carbon/carbides in the second case. However, as shown by chemical analysis, the total content of the oxygen and carbon did not exceed 0.2 wt.% and 0.08 wt.%, respectively. In the case of the Al additions, the obtained Ti-Al materials had more complicated phase compositions, depending on the amount of aluminum used. The porous material produced using the TiH_2_ + 20%Al powder blend had a two-phase α-Ti and Ti_3_Al composition (Figure 4, curve 1). The increase to 30% aluminum content caused the formation of a three-phase composition, namely, α + Ti_3_Al + TiAl (Figure 4, curve 2). Evidently, a short (0.5 h) sintering regime is not sufficient to achieve complete uniformity of the local composition of sintered Ti-Al materials. 

### 3.2. Compression of the Porous Materials

#### 3.2.1. Quasi-Static Tests

**Figure 5:**
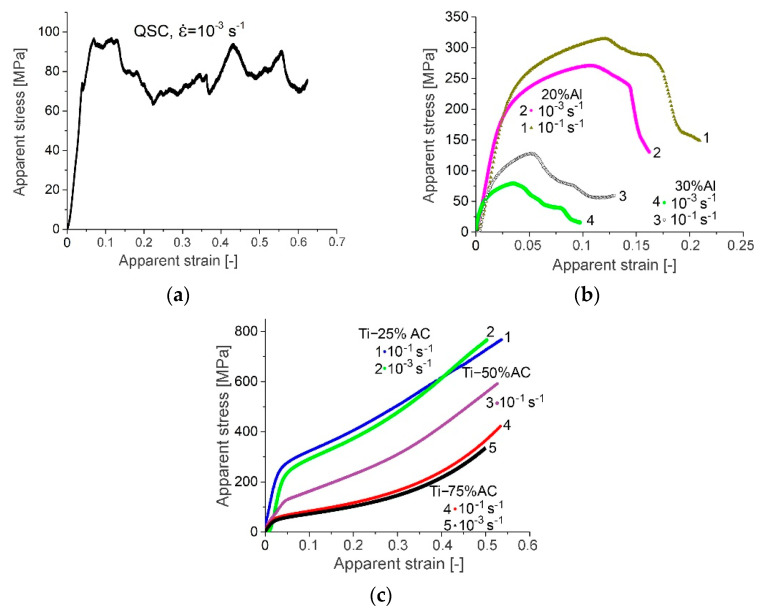
Quasi-static compression stress–strain curves of porous materials obtained using: (**a**) NaCl, (**b**) aluminum powder, and (**c**) ammonia carbonate (for two strain rate levels of 10^−3^ s^−1^ and 10^−1^ s^−1^).

The nominal (apparent) stress–strain curves of the porous samples were obtained with strain rates ranging from 10^−3^ to 10^−1^ s^−1^. The representative stress–strain curves for produced porous materials under quasi-static loading are presented in Figure 5. A comparison of these curves clearly indicates that the mechanical behavior of studied materials is rather different. The CP-Ti samples made with NaCl were characterized by a rather small value of plastic flow stress (be-low 100 MPa), and, despite the high cracking strain (above 60%), demonstrated very unstable plastic flow (stress fluctuation) during deformation (Figure 5a). Visually, during the test, this was accompanied by periodic abrupt “folding” of the sample, which could be associated with the high porosity and the resulting inhomogeneity in the spatial distribution of pores and bridges between them. Such a behavior was rather similar to behaviors observed in honeycomb structures built with Laser Engineering Net Shaping technology, using powders of Ti-6Al-4V alloy [20] or titanium powders and having 50 and 60% porosities [21]. 

The mechanical behaviors of the samples produced with 20% aluminum powder were characterized by a distinctly higher yield strength than the mechanical response of the CP-Ti made with NaCl, but they had essentially lower cracking strains (curves 1 and 2 in Figure 5b). The evident reason for such mechanical characteristics of the Ti-20%Al material was the presence of relatively brittle Ti_3_Al titanium aluminide in it (see Figure 4, curve 1). An increase in the content of aluminum powder in the initial mixture to 30% led to a dramatic deterioration in the mechanical characteristics of the sintered material (curves 3 and 4 in Figure 5b), which was undoubtedly caused by the formation of the second brittle TiAl intermetallic compound, in addition to Ti_3_Al (curve 2 in Figure 4).

Porous titanium obtained with ammonia carbonate (AC) showed more promising results (Figure 5c). In most cases, the stress–strain curves were smooth and typical for porous materials, i.e., a linear-like elastic stage appeared initially up to the yield stress level, followed by a period of long strain hardening, collapsed porous regions, and—in the case of Ti-50%AC and Ti-75%AC materials—an onset of densification. However, the stress–strain curve profile of the Ti-25%AC material was similar to the compression curve of the solid material (Ti-25%AC yield point was 232 MPa and it was lower by approximately 23% than the yield strength of commercially pure titanium), whereas other materials exhibited mechanical behaviors similar to open-cell foams (type I) or additively manufactured regular cellular materials [22]. At this point, it should be noted that the relative densities of Ti-50%AC and Ti-75%AC were 0.51 and 0.43, respectively; thus, they were higher than the upper value of the relative densities attributed to foams (according to the work [23]). Foam is defined as a cellular material, whose relative density ranges from 0.01 to 0.3). Hence, no plateau stage was observed, wherein stress was relatively constant over a large strain range. Instead, an increase in nominal stress was observed, which was associated with the effect of the strain hardening of the base material. Nevertheless, the visible positive slope of the nominal compression curve after exceeding the yield point was not only the effect of strain hardening but also the increase in the compression force, resulting from the increase in the specimen’s cross-section, especially in a material with low porosity, such as Ti-25%AC.

Noteworthy is the surprisingly high strain rate sensitivity of Ti-20%Al and Ti-30%Al in the quasi-static range. Porous materials made with the use of AC behave typically under quasi-static deformation conditions, i.e., they show a small effect of strain rate sensitivity.

Based on the above data, only two types of materials samples were selected for dynamic tests—the 2nd and 3rd types, i.e., produced with Al and AC powders (Table 1). Samples of the 1st type were not qualified for further studies not only because of their extremely unstable and poorly repeatable results obtained during quasi-static tests but also because of their extremely troublesome manufacturability, leading to intense contamination of the vacuum equipment by remnants of NaCl. 

#### 3.2.2. Dynamic Impact Compression Tests 

**Figure 6:**
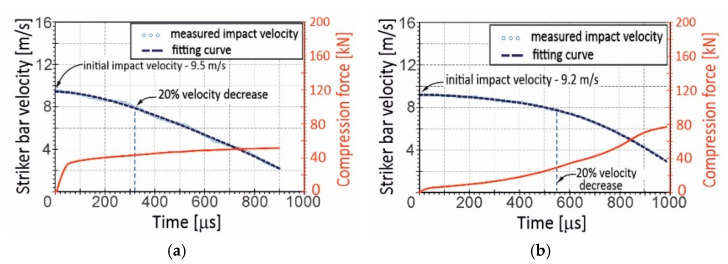
History of impact velocity and the compression force during the specimen crushing process of: (**a**) Ti-25%AC—A material with the lowest porosity; and (**b**) Ti-75%AC—A material with the highest porosity. Initial impact velocities were (**a**) 9.5 m/s and (**b**) 9.2 m/s.

The first series of dynamic experiments using the DIHPB technique was carried out at the impact striker bar velocity of 9.4 m/s on average. It was assumed that this level of initial impact velocity would ensure the achievement of equilibrium in the stress state in the dynamically deformed specimens. Unfortunately, the impact tests revealed that the kinetic energy of the striker bar was so low that the compression velocity of the specimens was distinctly inconstant. This referred especially to the Ti-20%Al and Ti-25%AC specimens, for which a 20% decrease in the compression velocity corresponded to the 0.19 relative shortening, whereas the Ti-75%AC specimen’s shortening achieved 0.49. For the purposes of comparison, Figure 6 represents changes in compression velocity for the Ti-25%AC specimen (the lowest porosity—Figure 6a) and for Ti-75%AC (the highest porosity—Figure 6b) against the background of the compression force curves. As also shown in Figure 6, the velocity decreases continuously. Such a phenomenon occurred not only due to the reaction force of the material specimen but also due to small velocity jumps resulting from the propagation of the stress wave, which repeatedly reflected from the free end of the striker bar. As the velocity drop of the striker was very small, and the applied measuring method was not enough sensitive to detect subtle changes in velocity, it could be assumed that the compression speed curves had a continuous nature. 

**Figure 7:**
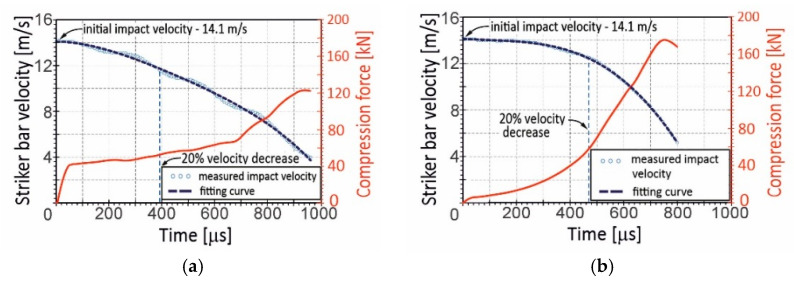
History of impact velocity and the compression force during the specimen crushing process at an initial impact velocity of 14.1 m/s on average for: (**a**) Ti-25%AC—a material with the lowest porosity; (**b**) Ti-75%AC—a material with the highest porosity.

To ensure that the specimen compression velocity under DIHPB conditions was relatively constant, the initial impact velocity was increased to 14.1 m/s on average (Figure 7). As a result, the relative shortening of the specimens for a 20% drop in the impact velocity was higher and amounted to 0.38 and 0.64 for Ti-25%AC and Ti-75%AC, respectively. Thus, in the case of the Ti-75%AC material, the specimen crushing took place at a relatively constant velocity for more than half of the entire deformation process.

**Figure 8:**
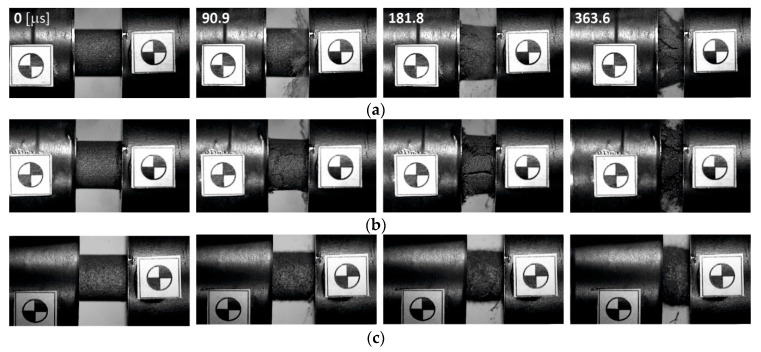

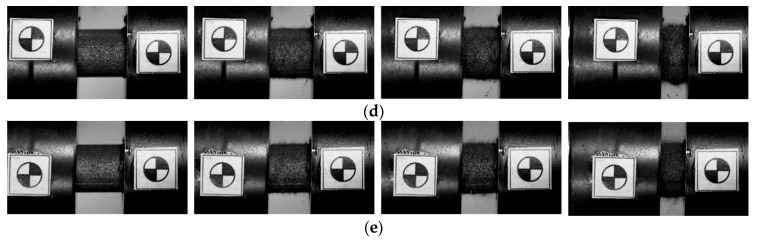
High-speed video frames of tests at 14.1 m/s for porous samples sintered using: (**a**) 20%Al, (**b**) 30%Al, (**c**) 25% AC, (**d**) 50%AC, and (**e**) 75%AC.

The presentation of the results, illustrating the mechanical behavior of the porous titanium specimens under impact conditions, began with a set of selected video frames collected in Figure 8. It is clearly visible that specimens obtained with the use of 20%Al begin to crack and brittle at intermediate stages of deformation (3rd and 4th frames in Figure 8a), whereas, on the side surface of the Ti-30%Al specimens, the first longitudinal cracks appeared after about 80 ms from the beginning of the crushing process (2nd frame in Figure 8b). In addition, the specimen’s deformation appears macroscopically inhomogeneous under dynamic loading, i.e., deformation and cracking front propagation is observed (see 3rd and 4th frames in Figure 8a,b), indicating a non-equilibrium stress state in the specimens.

In turn, the specimens prepared with the use of AC were characterized by more apparent plastic behavior, i.e., crack initiation and the formations of specimen fragments were not observed. In addition, porous Ti specimens sintered using AC initially deformed uniformly and then underwent the barreling effect. The exception being the Ti-75%AC material specimens, which barreled only after a long stage of compaction when they reached the level of full densification. This meant that the crushing history of the Ti-75%AC specimens was similar to the process of crushing cellular materials, during which the axial compression produced almost no lateral spreading; therefore, no clear barreling effect was noticed. However, according to [24], the lateral spreading of the porous Ti was greater during dynamic crushing compared to quasi-static because the mean Poisson’s ratio under dynamic loading was higher due to the more uniform deformation of the specimen.

**Figure 9:**
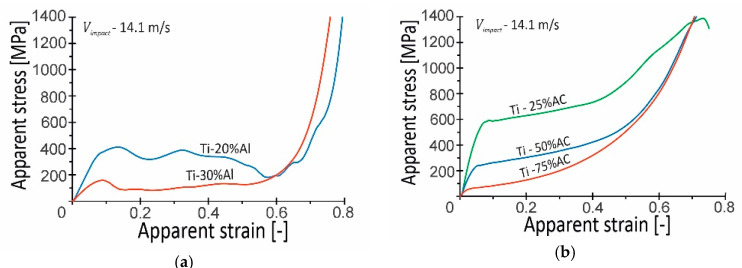
Representative dependencies between apparent stress and strain for porous specimens tested at initial impact velocity of 14.1 m/s: (**a**) Ti-X%Al; (**b**) Ti-X%AC.

The dynamic stress–strain dependencies for the porous specimens tested at a 14.1 m/s compression velocity are presented in Figure 9. The obtained compression curves are nominal and were calculated on the basis of the initial cross-section of the specimen. These representative stress–strain curves are derived from a dataset of at least three experiments, with a scatter of results less than 9% and 5% for the Ti-X%Al and Ti-X%AC materials, respectively. For the assumed level of initial impact velocity, i.e., 14.1 m/s, the corresponding average deformation rate is about 1300 s^−1^ for a 20% decrease in compression velocity after impact. The compression curves shown in Figure 9 are nominal and were calculated on the basis of the initial cross-section of the specimen. 

The brittle behavior of the Ti-X%Al samples was also confirmed by the profile of the dynamic compression curves (Figure 9a). After the initial deformation stage, during which the samples deformed elastically and plastically while maintaining the coherence of the material, there was a clear decrease in the compressive stress, which indicated the beginning of the specimens’ damage. Up to this point, the dynamic compression curve was smooth and typical. The failure of the specimen material occurred already at the apparent strains of 0.09 and 0.14 for Ti-20%Al and Ti-30%Al, respectively. However, a comparison with the quasi-static compression curves shows that the failure of the specimens under impact loading conditions occurred at slightly higher levels of deformation. The further stage of the specimens’ deformation took the form of crushing, during which large fragments of the respective specimen formed in the initial damage stage, were crushed, and then compressed. The compression curves corresponding to this stage were irregular with random characters, while the curves of the specimens made from porous titanium with the use of 30% Al were more smoothed. This was probably due to the higher brittleness and porosity of this material, which fractured at lower stress levels and in multiple locations simultaneously. As a result, the sample fracture process was relatively stable, i.e., without a step change in compressive stress. The final stage of specimen compression completed the crushing process, followed by the material densification stage. In both cases of the materials, densification of the fragmented specimens’ material occurred at a strain of about 0.6. 

A completely different history of deformation was revealed by tests carried out on porous titanium fabricated with the use of AC (Figure 9b). After the initial stage of compression, during which the samples deformed elastically, a slight decrease in stress occurred. From that moment on, the quasi-plateau phase began. Depending on the porosity, a different strain hardening effect was observed. The slope of the compression curves in the quasi-plateau range for the Ti-25%AC and Ti-50%AC specimens was almost the same, which indicated a similar degree of strain hardening, while the strain hardening of the material of the Ti-75%AC specimens was clearly smaller. Nevertheless, it should be noted that the slope of the compression curves (Figure 9b) in the part corresponding to the quasi-plateau phase was not only the result of the strain hardening of the specimen material but also the result of the increase in the cross-section of the specimen during compression. On the other hand, however, the slope of the curves of the Ti-75%AC samples with relatively high porosities of 57% was very similar in the initial stage of compression, and, after exceeding the strain value of 0.2, it started to increase, especially from the strain of 0.4. This different behavior of the Ti-75%AC material was probably due to the presence of air enclosed in the pores. As stated in [25], the gas effect was significant at the densification stage, wherein the crushing stress, i.e., the strain hardening effect, could be remarkably enhanced. This issue will be further addressed in Section 4.2 on the strain rate sensitivities of tested titanium-based porous materials. 

## 4. Discussion

### 4.1. Microstructures of Tested Samples 

**Figure 10:**
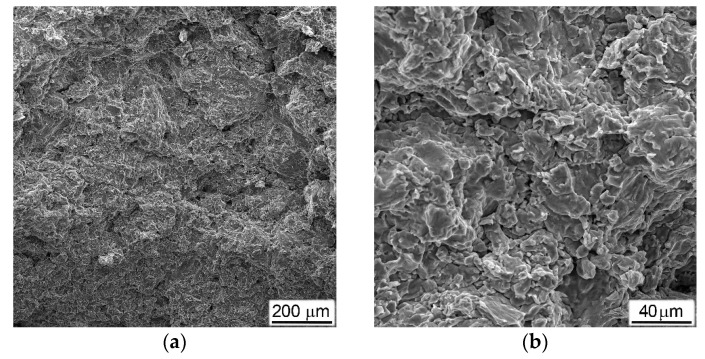

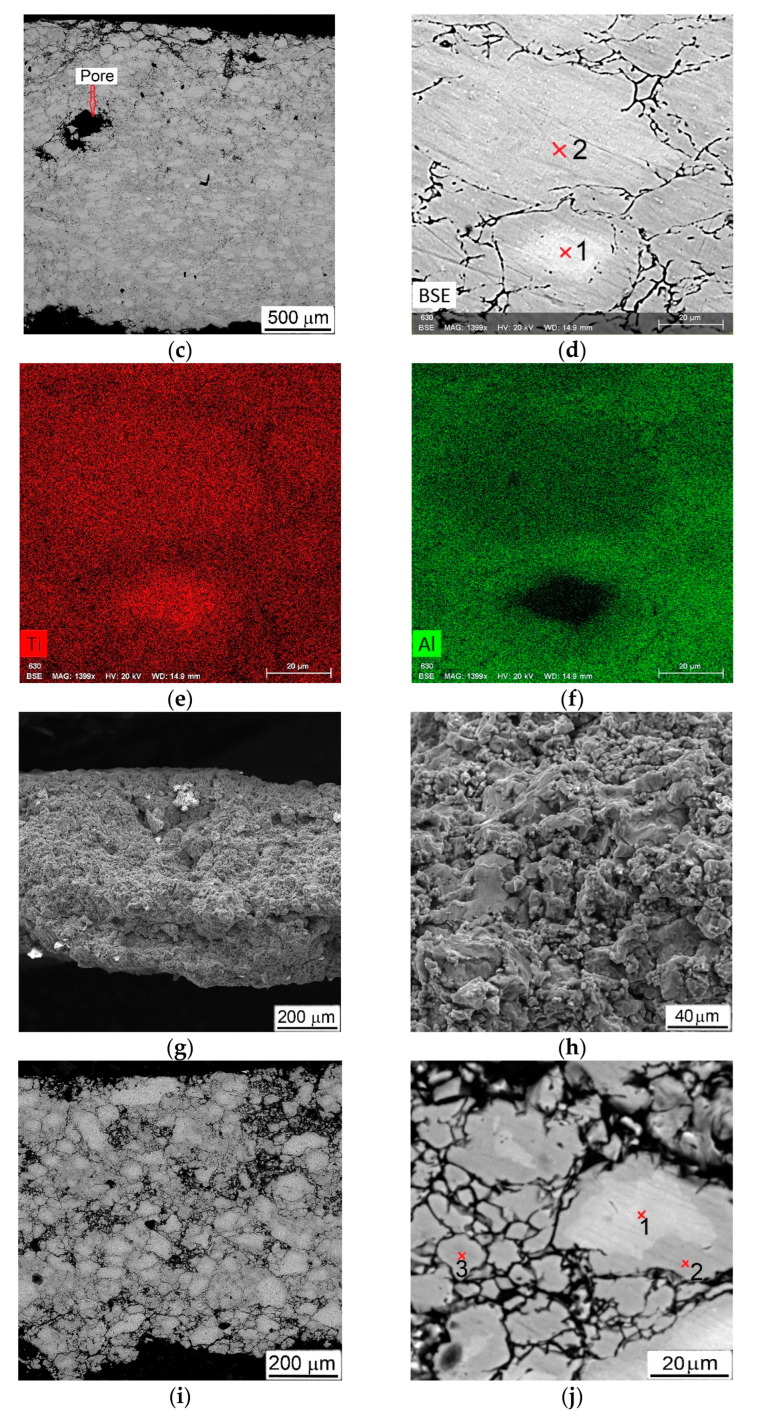
Fractured surfaces (**a**,**b**,**g**,**h**), microstructures (**c**,**d**,**i**,**j**), and chemical elements distribution maps (**e**,**f**) of porous samples produced with (**a**–**f**) 20%Al and (**g**–**j**) 30%Al subjected to DIHPB tests. SEM, (**a**,**b**,**g**,**h**) SE, (**c**,**d**,**i**,**j**) BSE. Points (1) and (2) on (**d**), as well as (1), (2) and (3) on (**j**) indicate the locations where the local chemical analysis was carried out. A description of the chemical composition at these points is given in the text below.

Typical examples of fractured surfaces (formed during DIHPB test or as a result of special breaking of the sample after testing), microstructures, and some results of chemical composition measurements for porous materials obtained with Al are presented in Figure 10. 

As in the mechanical behavior (Figure 5b and Figure 9a) and phase composition (Figure 4), the Ti-Al materials with various Al contents differed both in the nature of fractures and in their microstructures. In the material obtained with 20%Al, the fracture surface was characterized by the presence of small brittle facets (Figure 10a,b). At the same time, the microstructure demonstrated a rather dense material, in which only some of the largest primary pores were preserved, located closer to the edge of the sample (Figure 10c). The titanium base material was represented by tightly closing grains, somewhat flattened by the applied compressive load (Figure 10c). It should be especially noted that there was a difference in the contrast between the inner and outer parts of large grains in the image obtained of the backscattered electrons (Figure 10d). From the elements distribution maps (Figure 10e,f), it could clearly be seen that the lighter-looking inner parts of the grains were depleted in aluminum, while the outer parts of the same grains and the darker neighboring grains contained more aluminum and less titanium. The local measurements of the chemical compositions at points (1) and (2), indicated in Figure 10d, showed that the first of them had the composition 6.4 (wt.%)Al and 93.4%Ti, which corresponded to the solid solution of aluminum in titanium, and the second—14%Al and 86%Ti (or 23 (at.)%Al and 73%Ti)—which was close enough to the composition of the Ti_3_Al intermetallic. This result was in good agreement with the X-ray data (curve 1 in Figure 4). 

The results of a similar study on tested porous materials obtained with 30%Al are presented in Figure 10g–j. The surface of the fracture formed directly during testing, in addition to the previously noted brittle facets, and contained a large number of micro-fields that had the evident characteristics of melted metal (Figure 10g,h). Most likely, they were formed as a result of the melting of the Al particles during the heating for sintering, which, despite the fact that the molten aluminum immediately reacted with titanium (and, according to [26], underwent a whole series of transformations Ti + Al → TiAl_3_ → TiAl → Ti_3_Al), it still retained the morphological features of the melted metal. The backscattered electron image showed a huge number of crashed small grains, which did not have evident signs of plastic deformation, but bigger grains also had differences in contrast between the inner and outer parts (Figure 10i,j). Local chemical analysis (Figure 10j) showed that the chemical composition corresponded to Ti_3_Al (28 at.%Al + 72%Ti) in point (1), and in points (2) and (3)—47%Al + 53%Ti (i.e., closer to TiAl). This result mainly was in agreement with the X-ray data (curve 2 in Figure 4), except that the alpha phase of Ti was not found in the section field. 

**Figure 11:**
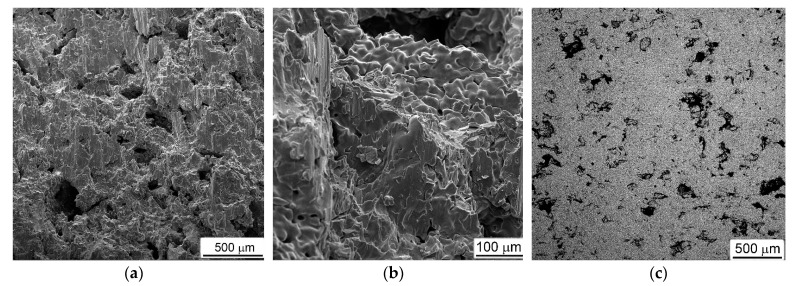

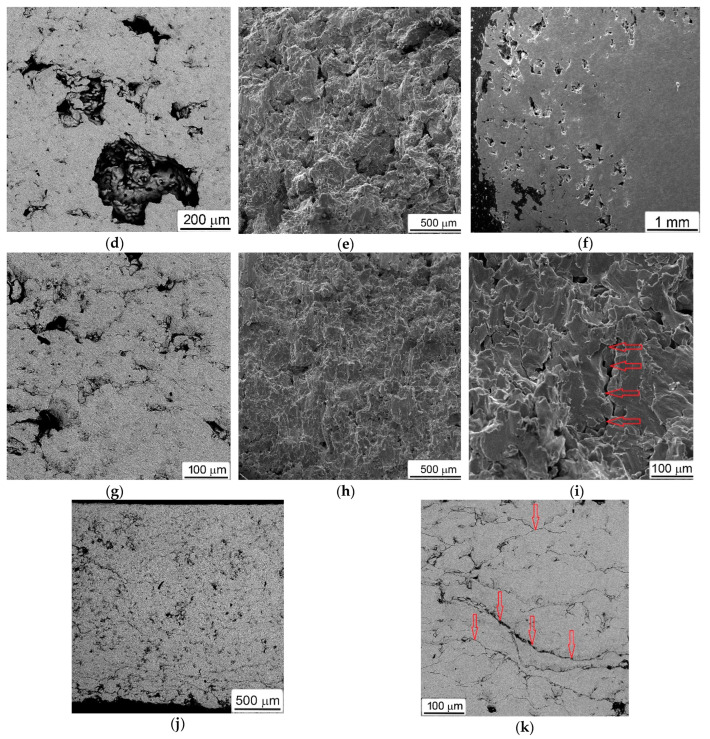
Fractured surfaces (**a**,**b**,**e**,**h**,**i**) and microstructures (**c**,**d**,**f**,**g**,**j**,**k**) of porous titanium produced with (**a**–**d**) 25%AC, (**e**–**g**) 50%AC, and (**h**–**k**) 75%AC subjected to DIHPB tests. SEM, (**a**,**b**,**e**,**h**,**i**) SE, (**c**,**d**,**f**,**g**,**j**,**k**) BSE. Arrows in (**i**,**k**) indicate boundaries of collapsed previous pores.

The situation observed in tested samples that were obtained with ammonium carbonate (Figure 11) differed fundamentally from the above. First of all, all fractured surfaces had a characteristic appearance of a viscous cut along the titanium bridges between the pores (Figure 11a,b,e,h,i). 

Between themselves, the fractures of materials obtained using different amounts of ammonium carbonate differed in the number of residual non-collapsed pores, which decreased at higher AC additions and, hence, had higher porosity levels (compare Figure 11a with Figure 11e,h). This circumstance could be explained by the fact that a material with a higher porosity had a lower resistance to the applied load and, thus, deformed to a greater extent. 

As for the chemical composition, all samples obtained using ammonium carbonate corresponded to commercially pure titanium with a total impurity content of no more than 0.3–0.4 wt.%. Moreover, most of the impurities (such as oxygen and carbon) were found not in the titanium matrix but were on the inner surface of the pores. 

### 4.2. Strain Rate Sensitivity 

**Figure 12:**
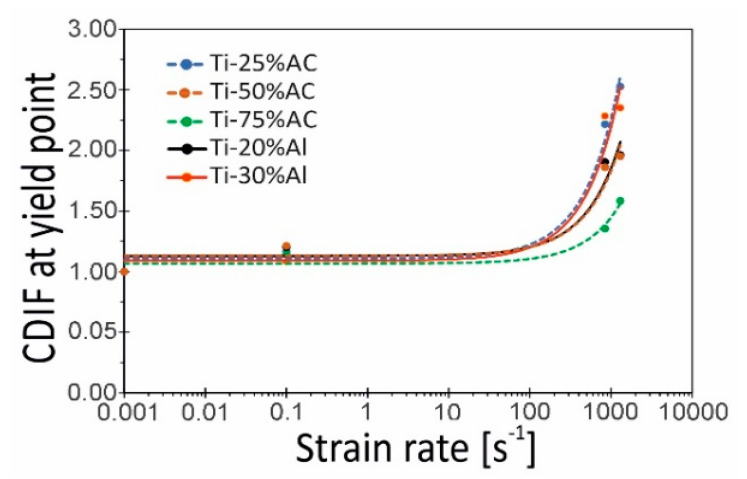
The compressive dynamic increase factor (CDIF) at yield stress versus the logarithm of strain rate for the tested porous materials.

In order to assess the impact of the strain rate on the compressive stress of the tested porous Ti variants, the Compressive Dynamic Increase Factor (CDIF) was used [27]. In the present work, it was defined as the ratio of dynamic stress at the yield point to corresponding quasi-static stress, representing the increase in stress under high strain rates. In Figure 12, changes in the CDIF value as a function of strain rate (0.001, 0.1, 840, and 1300 s^−1^) are shown. In general, all materials show a quasi-linear response between the logarithm of strain rate and yield stress up to strain rates of 1000 s^−1^. Within this strain rate range, the strain rate sensitivity of the tested porous materials is minor. However, a transition in strain rate sensitivity at rates above 10^3^ s^−1^ is observed. This behavior is expected and agrees with trends reported in the literature [22]. The highest sensitivity to strain rate, i.e., the highest CDIF values, was shown by the materials Ti-25%AC and Ti-30%Al being 2.53 and 2.35, respectively. In turn, the lowest CDIF of 1.58 was determined for Ti-75%AC. It is in line with literature reports [28], which state that the sensitivity to the strain rate of cellular material usually decreases with a decrease in relative density. 

**Figure 13:**
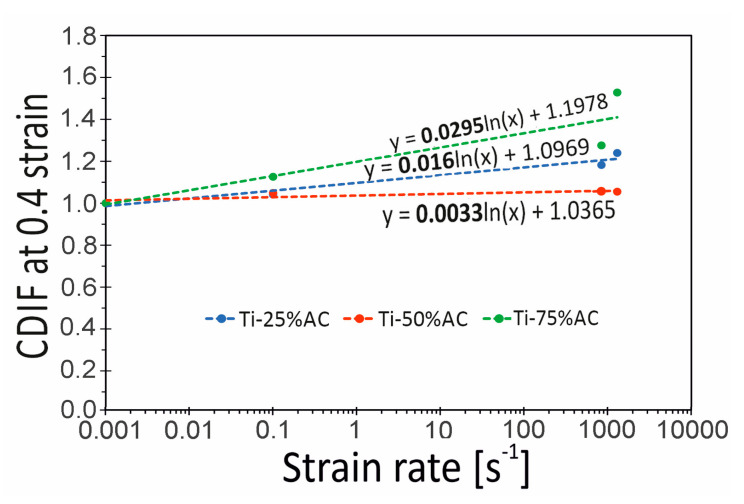
The compressive dynamic increase factor (CDIF) at a strain of 0.4 versus the logarithm of strain rate for the tested porous Ti-X%AC.

At this point, it should be emphasized that making a full assessment of the strain rate sensitivity of a given cellular material is a complex issue, and problems such as, for example, the influence of relative density on the strain rate sensitivity of metallic cellular materials remain unclear. For example, Figure 13 presents the Ti-X%AC curves illustrating the dependence of the CDIF parameter determined this time for strain 0.4. The data presented here show that the material with the lowest relative density (Ti-75%AC) exhibits the highest sensitivity to the strain rate in the range of high strain values. This contradicts the results of the analyses shown in Figure 12. As already mentioned above, the probable cause of the high sensitivity of Ti-75%AC to strain rate is the presence of gas trapped in the pores. The effect of entrapped air is particularly evident during the near-densification stage [25]. On the other hand, Ti-50%AC exhibits the lowest strain rate sensitivity, despite the fact that its relative density is lower than that of Ti-25%AC. This observation may, therefore, prove that the sensitivity to the strain rate of the base material plays a dominant role in the enhancement of compressive stress [22]. 

### 4.3. Energy Absorption Characteristics

Two main quantities were used to evaluate the energy absorption properties of porous titanium, which were energy absorption per unit volume ($E_{ab}$) and specific energy absorption (*SEA*).

**Figure 14:**
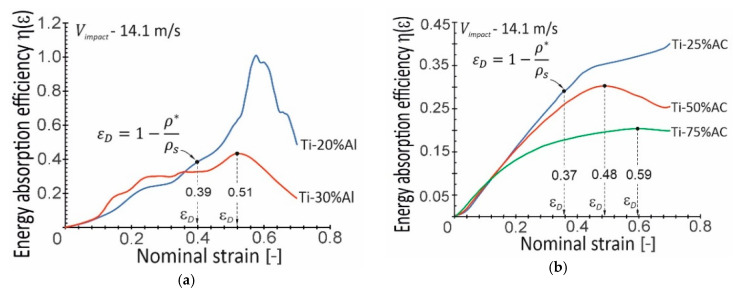
Energy absorption efficiency–strain curves of porous materials obtained using: (**a**) aluminum (Ti-X%Al); (**b**) ammonium carbonate (Ti-X%AC).

The mechanical energy absorption per unit volume ($E_{ab}$), also called energy absorption capacity, was defined as the energy necessary to deform a given specimen to specific strains. The value of $E_{ab}$ was expressed by the area under the stress–strain curve up to the densification strain $\varepsilon_{D}$, at which the plateau regime was finished. However, there were problems with determining the strain at the onset of densification for the tested porous Ti materials due to their ambiguous plateau regions. There were many approaches regarding the determination of the $\varepsilon_{D}$ value. Currently, the most common approach was to use energy absorption efficiency η(ε) as the mechanical energy absorbed up to a given nominal strain ε normalized by the corresponding stress value σ(ε) [29]. Then, the strain value of $\varepsilon_{D}$ corresponded to the maximum value of η(ε). Figure 14 shows the results of the value calculations $\varepsilon_{D}$. 

Unfortunately, in not all cases of the considered materials, it was possible to determine the value of $\varepsilon_{D}$ using the parameter η(ε). It resulted either from the unreliability of the obtained $\varepsilon_{D}$ the value resulting from the brittle behavior of the Ti-20%Al specimens during compression, or from the non-occurrence of the maximum of parameter η(ε) (Ti-25%AC). Therefore, in the above-mentioned cases of materials, the relationship proposed by Gibson and Ashby [23] and shown in Figure 14 was used to determine the value of $\varepsilon_{D}$. It should be noted that this approach was supported by the fact that the predictions of $\varepsilon_{D}$ values using the Gibson and Ashby formula for the remaining porous Ti materials were in good agreement with the results of analyses using the η(ε) parameter (see Table 2).

**Figure 15:**
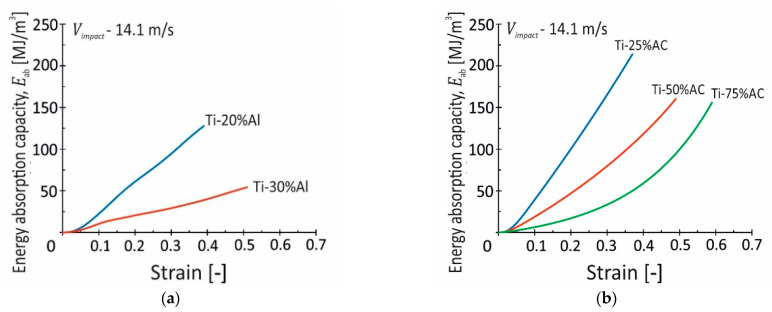
Energy absorption capacity–strain curves of Ti porous materials obtained using: (**a**) aluminum (Ti-X%Al); (**b**) ammonium carbonate (Ti-X%AC).

The energy absorption capacity–strain curves were calculated according to the dynamic stress–strain curves (Figure 9) in the determined range of densification strain and are shown in Figure 15. The curves of $E_{ab}$ show a linear or parabolic increase with the strain rising. The Ti-X%Al and Ti-25%AC materials reveal a near-linear dependence, while Ti-50%AC and especially Ti-75%AC are characterized by a parabolic relation between the $E_{ab}$ parameter and nominal strain. At the densification strain, the values of $E_{ab}$ are 128 and 54 MJ/m^3^ for the Ti-20%Al and Ti-30%Al materials, respectively, whereas they are 214, 160, and 156 MJ/m^3^ for Ti-X%AC, respectively. These results prove that the porosity and fracture mechanisms play a very important role in the energy absorption property. In the case of porous Ti materials fabricated with Al, their energy absorption capacities decrease as the porosity reduces. The same dependence is observed for the Ti-X%AC specimens, however, the level of $E_{ab}$ is higher compared to Ti-X%Al. In that case, low $E_{ab}$ values result from the high susceptibility of these materials to brittle fracture. The Ti-X%Al specimens undergo fracture at relatively early stages of plastic deformation by means of breakage of the titanium “bridges” between the neighboring pores. In the case of specimens, the Ti-20%Al specimen damage occurs locally but not simultaneously in the entire specimen volume due to the relatively small number of “bridges” in the titanium matrix. In the case of the Ti-30%Al specimens, despite the bigger amount of the titanium matrix, their “bridges” are more brittle due to the presence of non-ductile phases of titanium-aluminum intermetallic compounds. The completely different behaviors of the Ti-X%AC specimens are observed despite their even greater porosities (57% in the Ti-75%AC material). The titanium matrix remains plastic until the porous structure is fully densified.

**Figure 16:**
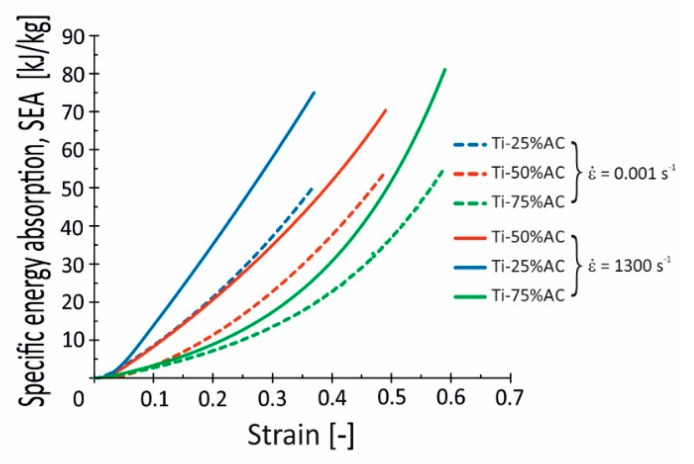
Specific energy absorption–strain curves of Ti-X%AC materials obtained under quasi-static and dynamic loading conditions.

Interesting results are also provided by the set of curves collected in Figure 16, where the results of specific energy absorption *SEA* calculations based on data obtained from quasi-static and dynamic experiments are presented. The *SEA* parameter reflects the energy absorption capacity of the specimen and is an important index for evaluating the energy absorption components. As presented in Figure 16, Ti-75%AC achieves the highest values of SEA, equal to 55 and 81 kJ/kg, which were calculated from quasi-static and impact test data, respectively. 

As also seen in Figure 16, large differences exist between quasi-static and dynamic *SEA* curves. This observation indicates a distinct enhancement of the considered porous titanium’s ability to absorb mechanical energy under dynamic loading conditions. In that regard, the Ti-25%AC and Ti-75%AC reveal the largest *SEA* increases of around 47%, while the increase was around 30% for Ti-50%AC. It supports the above-mentioned statements regarding strain rate sensitivity (see Section 4.2), as the strain rate sensitivity of the base material plays a dominant role in enhancing the absorption property of this porous material in the case of Ti-25%AC, while the high *SEA* value of Ti-75%AC is related to the phenomenon of gas trapped in the pores. 

### 4.4. Damping Properties

**Figure 17:**
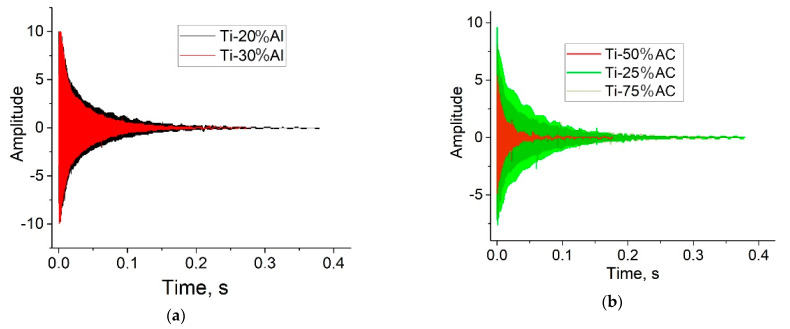
Comparison of damping curves of shock–sound vibrations in porous materials obtained with (**a**) Al, and (**b**) ammonia carbonate.

Interesting information on the examined porous materials was also obtained by measuring the attenuation of sound vibrations in the studied samples by the Resonance-Frequency-Damping Analysis method (Figure 17, and Table 3). The general trend for both types of samples is that the resonant frequencies and Young’s moduli decrease, while the damping abilities and loss rates increase as the pore-forming component increases. As with the properties discussed above in Section 4.2, Section 4.3 and Section 4.4, the material obtained with ammonium carbonate has an advantage over that obtained with aluminum in damping properties (Compare Figure 17a,b, and the data of Table 3). However, it should be noted that there is one exception to this regularity, namely, in the Ti-50%AC sample, the two parameters being, namely, the resonant frequency and the loss value, demonstrating extremal values relating to two other compositions.

### 4.5. Deformation Mechanism 

**Figure 18:**
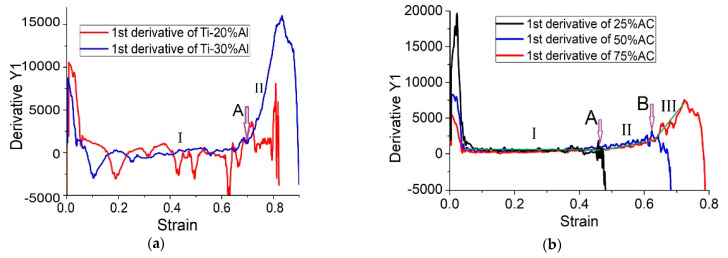
The result of differentiation of the deformation curves shown in: (**a**) Figure 9a, (**b**) Figure 9b. (I), (II), and (III) indicate different stages of samples’ plastic deformation; (A) and (B) show transition points from one stage to the next.

It is generally accepted that absorption energy depends strongly on porosity [30]. If the porosity is too high, the foam material crushes before the impact energy is sufficiently absorbed. If the porosity is too low, the stress in the foam exceeds the given critical value of the low absorbed energy. The high porous materials have maximum efficiency at the easy deformation stage, which is well seen in Figure 9a,b. As it was mentioned above, the stress–strain curves for the Ti-X%Al samples are characterized by unstable non-linear dependencies on the stage of deformation, related to the collapsing of pores (Figure 9a). The transformation of these curves made by taking the 1st derivative made the instability of the deformation process more visible (stage I in Figure 18a). Only in the Ti-30%Al specimens can a change in the nature of deformation after the inflection of the curve at point (A), when the material is compressed, in which the pores have completely collapsed, can be noted. 

Preliminary analysis of the curves in Figure 9b showed that three characteristic areas can be distinguished on them, which differ in conditional inflection points and inclination angles. This fact becomes more evident when transforming these curves by taking the derivative (Figure 18b). In all specimens, there is a horizontal linear section (I), which corresponds to a simple collapse of the pores. For the Ti-25%AC specimen, this process completes the entire deformation of the material. For the two other materials (Ti-50%AC and Ti-75%AC), there is a segment (II) that probably corresponds to the simultaneous collapse of the remaining pores and the dynamic hardening of the titanium matrix. Only in the Ti-75%AC specimens with the highest initial porosities there is one more section (III), that most likely corresponds to the dynamic hardening of completely densified titanium, in which all the pores have already been collapsed at the previous deformation stages. 

Three stages of porous material deformations were also described in [26,31]. The first was the stage of easy deformation, which made a major input into the damping efficiencies of the porous materials. The second and third stages were designated as the stages of easy and strong deformation strengthening, respectively. 

**Figure 19:**
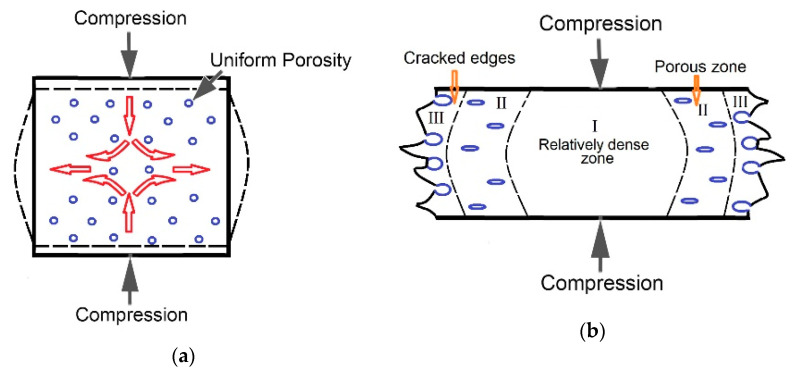
Schemes illustrating changes in the structure of ductile specimens (prepared with ammonia carbonate): (**a**) initial state and acting forces, (**b**) formation of non-uniform porosity after compression. (I), (II), and (III) at (**b**) indicate main zones that differ in microstructure/final porosity formed in the sample on dynamic compression.

The general scheme of the deformation process at the macro level during the compression of porous specimens is presented in Figure 19. At the initial stages of deformation (corresponding to segments (I) and (II) in Figure 18), especially by QSC, the beginning of the plastic flow in certain directions (as it is indicated by the red arrows in Figure 19a) and a barrel formation took place. During this stage of deformation, the uniform, albeit partially “flattened”, porosity is retained in the specimen. However, as the deformation process develops and the pores collapse in the central part, where the plastic flow is maximum, residual porosity is still observed. At the distance from the center, where the degree of deformation is smaller, at the specimen edges, the pores partially open, creating surface cracks (Figure 19b). 

### 4.6. Comparison with Other Porous and Cellular Metallic Materials

Firstov and Podrezov proposed an approach of optimizing porous structures for reaching the maximum values of work hardening based on a comparative analysis of the metallic base and pore contributions [31]. Their approach allowed us to find the highest correlations between the maximum value of deformation energy under compression and the relative porosities for aluminum and nickel foams as 4.08 MJ/m^3^ for 0.71 (71%) and 13.5 MJ/m^3^ for 0.68 (68%), respectively. The comparison of aluminum and nickel foams, with results on Figure 9, showed the superiority of our material by at least one order of magnitude. 

According to [21], the maximum compressive strength for porous Ti produced with NaCl as a pore holder monotonically decreased from 450 MPa to 130 MPa, with an increase in porosities from 35% to 60%. The authors of [20] obtained a similar dependence between maximum values of absorbed energy and relative porosity for the honeycomb structure of Ti-6Al-4V, which decreased from 322 J to 120 J, with a decrease in relative density (the same as increase in relative porosity) from 0.36 to 0.23. Comparing our above presented results, we can conclude that porous titanium produced with ammonia carbonate (see Figure 12 and Figure 13) had characteristics not worse than the cellular honeycomb structures manufactured by LENS technology from Ti-6Al-4V powder, in terms of maximum values of absorbed energy [20]. Moreover, our porous material is much cheaper, considering the cost of the initial powders and the complexity of the manufacturing technology. 

## 5. Conclusions

The mechanical behavior of the porous titanium and Ti-Al materials prepared via three different approaches was studied under quasi-static and dynamic compression loading conditions. The influence of porosities, phase composition features, and microstructures on the mechanical behaviors of materials were analyzed. Based on the experimental data presented and their analysis, the following conclusions could be drawn:

1. Among the considered methods of manufacturing porous materials, the most promising is the use of ammonium carbonate as a pore holder. This method is quite simple and technologically convenient, providing controlled porosity and desired mechanical characteristics. Porous Ti-Al materials with fewer promising characteristics were produced using a reaction of molten Al with titanium particles. The worse characteristics of the sintered porous titanium were obtained using NaCl crystals as a space holder material.
2. The mechanical behavior under quasi-static and dynamic compression of porous titanium materials obtained by various methods was determined by two main factors: (a) the porosity value and (b) the presence of additional phases, such as titanium aluminides in the case of Ti-Al materials. Single-phase (h.c.p.) porous titanium produced with ammonium carbonate demonstrated a mechanical behavior that was similar to a solid metal if the material porosity was lower than 50%. Otherwise, the tested Ti porous materials behave similar to metal foams. In the case of porous Ti-Al materials, the presence of brittle intermetallic phases led to a sharp decrease in plasticity and material fracture at the beginning stages of deformation. The higher the content of aluminum used for pore formation, the greater the number of intermetallic compounds formed and the earlier and more extensively cracking of the material.
3. It was found that porous titanium materials were sensitive to the strain rate when the latter exceeded 10^3^ s^−1^. The highest sensitivity to the strain rate was shown by the materials prepared using aluminum. Specimens prepared with ammonium carbonate were much less sensitive to the strain rate. However, the dependence of the strain rate sensitivity on the porosities of these specimens was ambiguous, probably due to the effect of the presence of gases trapped in the pores.
4. The porous titanium matrix produced using ammonium carbonate demonstrated plastic deformation behavior until the porous structure was fully densified, allowing the accumulation of essentially more mechanical energy, possessing higher energy absorption capacity. In contrast, the cracking of the brittle Al-Ti intermetallic bridges between the pores at relatively early stages of compression resulted in a relatively low impact energy absorption capacity.

On the basis of the entire complex analysis of the obtained results, a scheme was proposed that described the mechanism of deformation of the synthesized and studied samples of porous titanium. As the plastic deformation progressed, the following successive three stages could be observed: (1) a collapse of the pores; (2) plastic deformation of the titanium bridges in the Ti-X%AC material, or their destruction in the Ti-X%Al material; and (3) further plastic deformation with the dynamic hardening of completely densified titanium (observed only for the Ti-X%AC material).

## Figures and Tables

**Table 1:** Some features of produced porous titanium.

	Weight % of Pore Holder, or Pore Forming Substance	Vacuum Sintering Temperature, °C	Sintering Exposure, Hours	Porosity, %	Average Density ρ*, g/cm^3^	Relative Density $\frac{\rho *}{\rho_{s}}$
CP-Ti, Type #1, NaCl as space holder						
1	60	1000 + 1200	0.5 + 2	71	1.31 ± 0.02	0.29
Ti-Al, Type #2, Al for pores formation						
2	20	1000	0.5	35	2.729 ± 0.037	0.65
3	30	1000	0.5	42	2.244 ± 0.054	0.58
CP-Ti, Type #3, ammonia carbonate [(NH_4_)_2_CO_3_] as space holder						
4	25	1250	2	37	2.850 ± 0.026	0.63
5	50	1250	2	49	2.281 ± 0.034	0.51
6	75	1250	2	57	1.922 ± 0.019	0.43

$\rho_{s}$—density of solid titanium—4.5 g/cm^3^, Ti + Al alloys in the solid (not porous) states have following densities: Ti-20%Al 4.2 g/cm^3^, Ti-30%Al 3.9 g/cm^3^.

**Table 2:** The values of the strain at the onset of densification $\varepsilon_{D}\;$ for the tested porous Ti materials, determined with different approaches.

	Ti-20%Al	Ti-30%Al	Ti-25%AC	Ti-50%AC	Ti-75%AC
$\mathrm{Value}\;\mathrm{of}\;\varepsilon_{D}$ from η(ε)	-	0.51	-	0.49	0.59
$\mathrm{Value}\;\mathrm{of}\;\varepsilon_{D}$ from Gibson & Ashby formula	0.39	0.50	0.37	0.48	0.57

**Table 3:** Damping measurement results of porous Ti materials.

Material	Resonance Frequency (Hz)	Loss Rate (%)	Damping Ability	Young Modulus (GPa)
Ti-20%Al	4926	16	1	44
Ti-30%Al	4411	24	1.7	27
Ti-25%AC	5523	17	1.8	45
Ti-50%AC	4187	60	2.7	18
Ti-75%AC	4284	14	2.7	16

## Data Availability

The data are available from the authors upon request.
